# Characterization, isolation and cloning of sugarcane genes related to drought stress

**DOI:** 10.1186/1753-6561-8-S4-P110

**Published:** 2014-10-01

**Authors:** Larissa Mara Andrade, Thiago Romanos Benatti, Paula M Nobile, Maria Helena Goldman, Antonio Figueira, Ana Lilia Alzate Marin, Michael dos Santos Brito, Jorge da Silva, Silvana Creste

**Affiliations:** 1 Faculdade de Medicina de Ribeirão Preto/ Centro de Cana-IAC, Campinas, Brazil; 2 Texas A&M Agrilife Research and Extension Center, Dallas, TX, USA; 3 LaFiMP - Depto. de Biologia Vegetal - IB - UNICAMP/ Centro de Cana-IAC, Campinas, Brazil; 4 Faculdade de Filosofia de Ribeirão Preto, Brazil; 5 Centro de Energia Nuclear na Agricultura, Piracicaba, Brazil; 6 Faculdade de Medicina de Ribeirão Preto, Brazil; 7 Centro de Cana- IAC, Campinas, Brazil

## Background

Sugarcane is a major crop worldwide as raw material for sugar and ethanol production. Drought is one of the most limiting factor that affects sugarcane productivity. In order to understand the mechanisms of drought response, field and greenhouse assays were conducted with two drought-contrasting sugarcane genotypes (IACSP94-2094/tolerant and IACSP97-7065/sensitive), and several genes up/down-regulated under drought stress identified by microarrays and RNAseq analyses. Ten differential expressed genes in both assays were evaluated by qPCR [1], and three of them showed the transcriptional profile related drought tolerance: a- Lipoxygenase (*ScLOX*), acting in the biosynthesis of the jasmonic acid precursor, and recent studies showed their role in defense against drought stress [3]; b- Dehydrin, correlated to drought stress and associated to maintenance of turgor cells, [2]; c- Dirigent-jacalin, associated to resistance disease and abiotic stress tolerance [4] and also related to jasmonic acid, an important hormone on plant defense. These genes were chosen as target for functional analyses in rice and sugarcane transgenic plants.

## Methods

The full-length sequences of the coded sequences of ScLOX, dehydrin and dirigent-jacalin genes were accessed by SMARTer RACE cDNA Amplification Kit (Clontech) using the tolerant genotype IACSP94-2094 mRNA. The cloning was performed using the vector pGEM-T Easy (Promega) and *E. coli *DH10B lineage. The clones were sequenced. After, the sequences were subcloned into pDONR 211 gateway vector and subsequently cloned in the overexpression and silencing vectors constructions to transformation plants via *Agrobacterium*.

## Results and conclusions

The coding sequence of *ScLOX *is incomplete at SUCEST (Sugarcane Functional Genomics Database). After many attempts of full-length transcripts *ScLOX *amplifications, only 3'RACE fragment from five clones were identified as the tag gene. On the other hand, the sequences from the 5'RACE amplification clones matched with others members from Lipoxygenase gene family. Nevertheless, the dehydrin and dirigent-jacalin were successful isolated and cloned as full-length coded sequences. From the 10 clones sequencing, it was found two allelic variant with a frameshift mutation and one as same identify of dehydrin sequences from the nucleotide databases queried. From 16 clones sequencing for dirigent-jacalin, it was found one allelic variant with frameshift mutation and one sequence matching the sequences from the database queried. The allelic variants, representing apparently nonfunctional alleles, were one of most difficulties in cloning the genes coded regions. This difficulty is attributed due the polyploidy and complexity genome of sugarcane. The allelic variant choice for the functional analysis was based on amino acid sequences alignment among sugarcane and related plants, e.g. rice, sorghum and maize, available in Phytozome and NCBI databases. The isolated coding sequences were engineered in plant overexpression and silencing construction vectors. Thereafter, rice and sugarcane embryogenic callus were transformed via *Agrobacteriumtumefaciens *and regenerated plants are under evaluation. The steps of identification, isolation and characterization of genes associated with response to stress are crucial for the development of transgenic plants improved for drought tolerance.
